# Intrinsic bifunctional photocatalytic water splitting and high carrier mobility in novel Janus SnXNH (X = S/Se/Te) monolayers: a first-principles prediction

**DOI:** 10.1039/d6ra04129a

**Published:** 2026-07-03

**Authors:** Pham T. Truong, Tran P. T. Linh, Le T. N. Tu, Tran N. K. Duy, Cuong Q. Nguyen, Nguyen T. Hiep

**Affiliations:** a Division of Physics, School of Education, Dong Thap University Dong Thap 870000 Vietnam; b Department of Physics, Hanoi National University of Education 136 Xuan Thuy Cau Giay Hanoi Vietnam; c Institute of Research and Development, Duy Tan University Da Nang 550000 Vietnam nguyenthihiep2@duytan.edu.vn; d Faculty of Natural Sciences, Duy Tan University Da Nang 550000 Vietnam

## Abstract

The pursuit of innovative materials for next-generation technologies has stimulated significant interest in two-dimensional Janus systems. Herein, based on density functional theory we propose and investigate physical properties of SnXNH (X = S/Se/Te) monolayers. Their crystal structures and dynamical stabilities are first evaluated. The computational results indicate that while SnSNH and SnSeNH are dynamically stable, SnTeNH is determined to be unstable. Consequently, only these two stable monolayers are subsequently examined in detail for their thermal, energetic, and mechanical stabilities, as well as their electronic, transport, and photocatalytic characteristics. The results confirm the thermal stability at room temperature of SnSNH and SnSeNH systems, along with strong energetic and mechanical robustness, indicating their feasibility for experimental synthesis. Electronic structure calculations reveal that SnSNH and SnSeNH are direct-bandgap semiconductors with bandgap energies ranging from 0.29 to 2.13 eV. In particular, SnSNH exhibits a suitable bandgap, making it a promising candidate for future exploration in photovoltaic applications. Carrier transport properties indicate that electron mobilities in both SnSNH and SnSeNH monolayers are significantly higher than those of holes due to their smaller effective masses. Moreover, the SnSNH monolayer demonstrates pronounced anisotropy in carrier mobility with respect to both transport direction and carrier type. From the large surface potential differences, the band edge positions of SnSNH and SnSeNH satisfy the requirements for water redox potential. Overall, these findings provide valuable insights that may inspire further exploration of novel two-dimensional Janus structures as efficient electronic and photocatalytic materials for technological applications.

## Abstract 2

The pursuit of innovative materials for next-generation technologies has stimulated significant interest in two-dimensional Janus systems.

## Graphical abstract

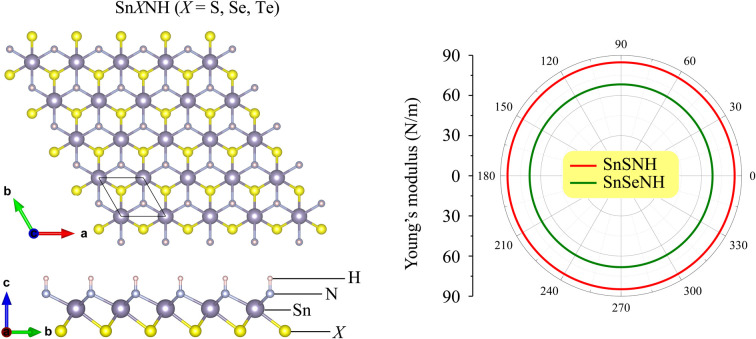

## 1 Introduction

The growing demand for high-performance materials in modern technologies has prompted extensive research into the discovery of novel material systems. In this context, two-dimensional (2D) nanomaterials have received considerable attention owning their unique structural characteristics and remarkable physical properties, which are highly advantageous for electronic and optoelectronic applications.^1–5^ Numerous 2D systems, including graphene, metal oxides, transition metal dichalcogenides (TMDs), phosphorene, and MXenes, have been widely investigated using both theoretical and experimental approaches.^6–9^ In recent years, Janus monolayers have been identified as a special class of 2D materials characterized by intrinsic structural asymmetry.^10^ This asymmetry eliminates inversion symmetry, leading to the emergence of an internal out-of-plane electric field and broken mirror symmetry, which fundamentally influence their electronic properties.^11^ As a consequence, Janus materials exhibit remarkable phenomena, including tunable electronic structures, Rashba spin splitting, and piezoelectricity.^12–14^ Notably, with high specific surface area, 2D Janus materials expose a high ratio of active sites to the surface, which enhance catalytic activity and improve charge separations for water splitting.

The formation of 2D Janus structures is commonly achieved by breaking the out-of-plane symmetry of a pristine monolayer through selective chemical modification.^15–17^ One effective strategy is the selective substitution of atoms in the top layer, where a parent 2D material is exposed to a controlled reactive environment, enabling the replacement of surface atoms on one side while preserving the opposite layer. For example, Janus MoSSe monolayers have been experimentally realized *via* post-synthesis chalcogen exchange from MoS_2_ using Se vapor,^10^ as well as through chemical vapor deposition techniques.^18^ Alternatively, surface engineering approaches, such as hydrogenation or halogenation, provide a versatile pathway to construct Janus structures by functionalizing only one side of the monolayer. In such cases, hydrogen or halogen atoms (*e.g.*, F, Cl) are selectively adsorbed onto a single surface, as demonstrated in theoretical and experimental studies of functionalized graphene and TMD-based Janus systems.^19,20^ These methods effectively break inversion symmetry and induce an intrinsic out-of-plane electric field, leading to novel electronic, optical, and catalytic properties. In this work, these two approaches are combined to construct SnXNH (X = S, Se, Te) monolayers, and their physical properties are explored based on the density functional theory (DFT) calculations. The stabilities of these systems are systematically assessed through elastic properties, phonon dispersion spectra, cohesive energy, and *ab initio* molecular dynamics (AIMD) simulations. Electronic structures are evaluated using both Perdew–Burke–Ernzerhof (PBE) and Heyd–Scuseria–Ernzerhof (HSE06) functionals to obtain reliable bandgap energies. Carrier transport behavior is investigated by calculating mobilities within the deformation potential formalism, considering effective masses and elastic moduli. Furthermore, band-edge alignment is analyzed to determine their potential applicability of these monolayers in photocatalytic systems. It is worth noting that the novelty of the proposed SnXNH monolayers extends beyond the construction of another Janus system. The combination of selective atomic layer substitution and surface hydrogenation in this design simultaneously enhances structural asymmetry and intrinsic out-of-plane polarization, leading to strong built-in electric fields that facilitate charge-carrier separation.

## 2 Computational methods

All calculations were performed within the framework of first-principles methods based on DFT, as implemented in the Vienna *Ab initio* Simulation Package (VASP).^21,22^ Structural optimization was conducted by allowing both lattice constants and atomic positions to relax until the Hellmann–Feynman forces acting on each atom were reduced below 1 × 10^−3^ eV Å^−1^, while the total energy difference between consecutive ionic steps was converged to less than 1 × 10^−6^ eV. The interaction between core and valence electrons was treated using the projector augmented-wave (PAW) formalism. The electronic wave functions were expanded in a plane-wave basis set with a kinetic energy cutoff of 500 eV. The Brillouin zone was sampled using a (15 × 15 × 1) *k*-point mesh constructed according to the Monkhorst–Pack scheme.^23^ To prevent artificial interactions between periodic images along the out-of-plane direction, a vacuum spacing of approximately 20 Å was introduced. Dispersion interactions arising from long-range van der Waals forces were incorporated *via* the DFT-D3 correction scheme.^24^ Charge transport characteristics were evaluated within the deformation potential (DP) theory framework,^25^ while band-edge positions were analyzed to determine the alignment relevant to electronic and photocatalytic behavior. It should be noted that the PBE functional was employed for structural relaxations and carrier mobility calculations because of its reliable description of band dispersion and computational efficiency. In contrast, the HSE06 hybrid functional was used exclusively to determine the absolute band-edge positions. This approach is necessary to mitigate the self-interaction error inherent in PBE and to provide a more accurate assessment of the thermodynamic driving forces for photocatalytic water splitting. The thermal stability of the systems was examined through AIMD simulations at 300 K over a time scale of 6 ps.^26^ Dynamical stability was further confirmed by calculating phonon dispersion spectra using the finite-displacement method, as implemented in the PHONOPY package with a 6 × 6 × 1 supercell.^27^ The exchange–correlation effects were initially described using the PBE functional within the generalized gradient approximation.^28^ For improved accuracy in predicting electronic properties, especially band gaps, additional calculations were carried out using the screened hybrid functional HSE06.^29^

## 3 Computational results and discussion

### 3.1 Geometric structures and stabilities

**Fig. 1:**
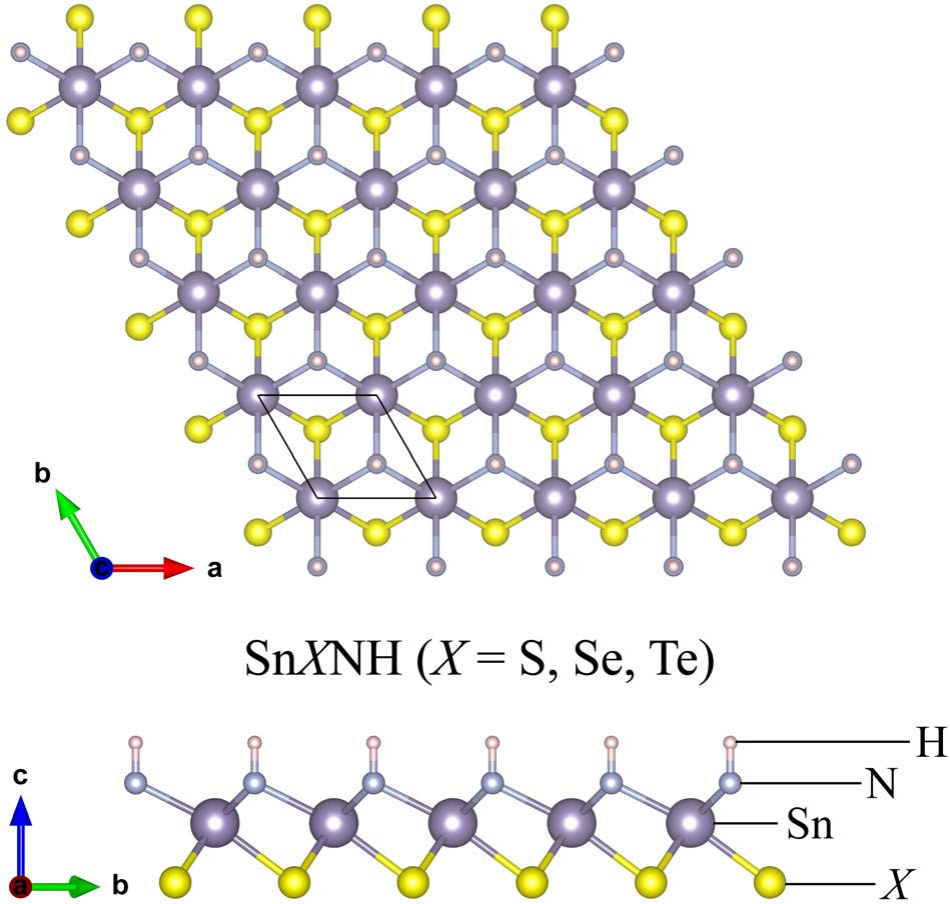
Crystal structures of SnXNH (X = S, Se, Te) monolayers from different views.

The optimized geometries of SnXNH monolayers, where X corresponds to the chalcogen atoms S/Se/Te, are illustrated in Fig. 1. All three structures exhibit a hexagonal arrangement belonging to the *P*3*m*1 space group. Each structure is composed of four atomic sheets, where a central Sn–N bilayer is enclosed by X and H layers. The calculated structural parameters in Table 1 indicate that the lattice constant increases systematically from 3.51 Å for SnSNH to 3.61 Å for SnSeNH and 3.64 Å for SnTeNH. Consistent variations are also observed for the Sn–X bond distances, which gradually expand with heavier chalcogen elements. As a result, the total thickness of the monolayers increases from 3.57 to 4.07 Å. This behavior can be attributed to the progressive enlargement of atomic size from S to Te,^30,31^ reflecting the structural adaptability of the SnXNH system.

**Table 1:** Lattice constants *a* (Å), *d*_Sn–X_, *d*_Sn–N_, and *d*_N–H_ (Å) bond lengths, monolayer thickness Δ*h* (Å), and cohesive energy *E*_coh_ (eV per atom) of SnXNH configurations

	*a*	*d*_Sn–X_	*d*_Sn–N_	*d*_N–H_	Δ*h*	*E*_coh_
SnSNH	3.51	2.53	2.27	1.03	3.57	−5.15
SnSeNH	3.61	2.65	2.29	1.03	3.67	−4.92
SnTeNH	3.64	2.97	2.30	1.03	4.07	−4.63

To elucidate the structural stability and bonding strength of SnXNH monolayers, we first calculated their cohesive energy (*E*_coh_). This fundamental quantity represents the required energy to disassemble a solid into its constituent isolated atoms; thus, a more negative value indicates stronger interatomic interactions and enhanced energetic stability. The cohesive energy of SnXNH (X = S, Se, Te) is defined as:1
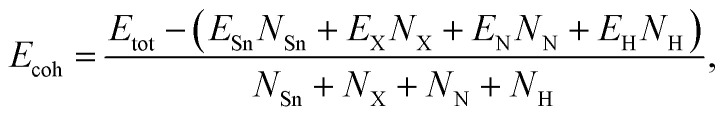
where *E*_tot_ is the total energy of the unit cell, *E*_Sn_, *E*_X_, *E*_N_, and *E*_H_ are the energies of the isolated Sn, X, N, and H atoms, and *N*_Sn_, *N*_X_, *N*_N_, and *N*_H_ represent their respective quantities within the unit cell.

As summarized in Table 1, the calculated cohesive energy of the SnSNH, SnSeNH, and SnTeNH monolayers are −5.15, −4.92, and −4.63 eV per atom, respectively. The clear trend toward less negative values from SnSNH to SnTeNH indicates a gradual reduction in binding strength as the chalcogen atom becomes heavier. In particular, the SnSNH monolayer exhibits the most negative *E*_coh_ value, implying the highest intrinsic stability among the studied structures. This enhanced stability can be attributed to its shorter bond lengths, which lead to more robust chemical bonding within the lattice. The obtained *E*_coh_ values of SnSNH, SnSeNH, and SnTeNH are comparable to those reported for several well-established two-dimensional materials, such as BGaS_2_ (−5.02 eV per atom),^32^ Si_2_SSe (−5.10 eV per atom),^17^ and STiSiP_2_ (−4.78 eV per atom).^33^ This result confirms that all three structures are energetically stable. As a first-principles theoretical investigation, determining the optimal specific experimental parameters (*e.g.*, precise temperatures or substrate choices) required to synthesize SnSeNH and prevent potential decomposition is beyond the scope of this work. Establishing these precise synthesis protocols remains an open and exciting challenge for future experimental studies.

**Fig. 2:**
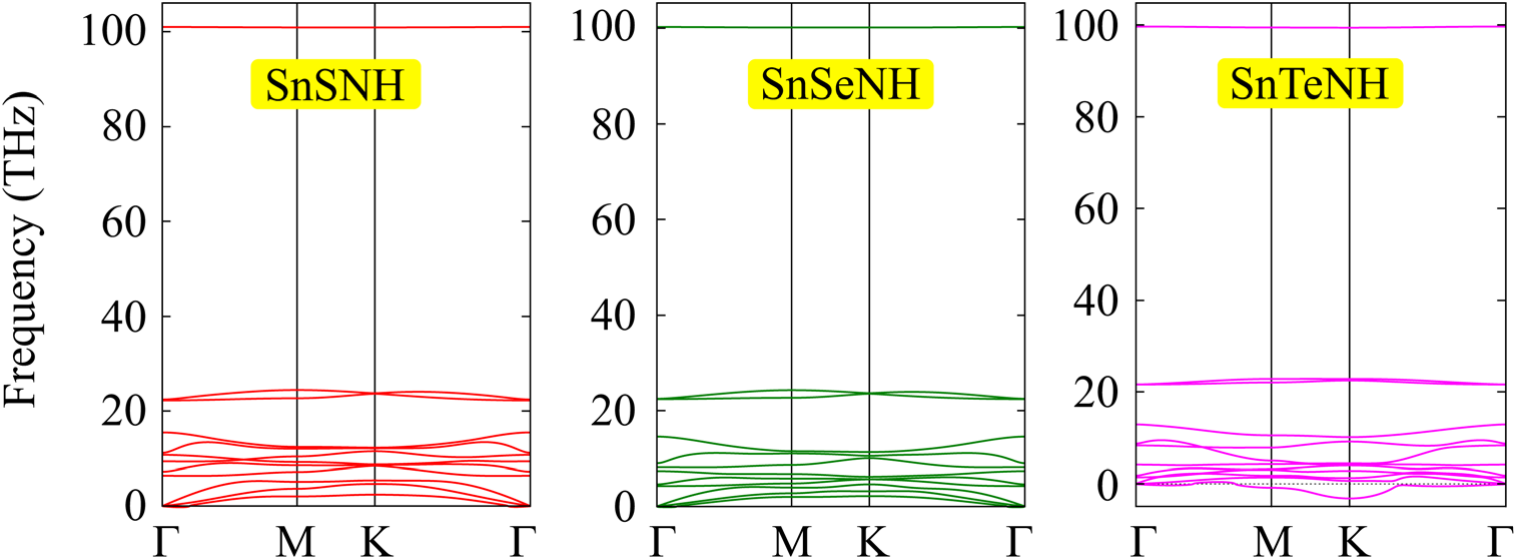
Calculated phonon dispersion curves for SnSNH, SnSeNH and SnTeNH monolayers.

More importantly, we perform phonon spectrum calculations for the SnXNH monolayers to further evaluate their dynamical stability. Phonon analysis is a crucial step in determining whether a crystal structure can sustain small atomic perturbations without undergoing structural distortion, as it provides direct insight into lattice vibrational modes across the entire Brillouin zone. A structure is considered dynamically stable only if all vibrational frequencies are real and positive. The absence of imaginary frequencies confirms the existence of sufficient restoring forces to counteract atomic displacements. In contrast, the presence of imaginary frequencies indicates that the restoring forces vanish, rendering the crystal structure dynamically unstable. Phonon spectra calculated along the *Γ*–*M*–*K*–*Γ* of BZ are presented in Fig. 2. Due to the presence of four atoms in the unit cell, the systems exhibit twelve phonon modes, including three acoustic and nine optical branches. For SnSNH and SnSeNH monolayers, no imaginary modes appear across the entire BZ, confirming the dynamical robustness of these systems. However, the phonon spectrum of SnTeNH exhibits imaginary frequencies near the K region of the Brillouin zone with a maximum magnitude of about 4 THz, indicating that the free-standing monolayer is dynamically unstable within the harmonic approximation. This result suggests a tendency of the lattice toward structural distortion rather than proving that the material is unsynthesizable. Based on this consideration, only SnSNH and SnSeNH monolayers are further examined in the next section.

**Fig. 3:**
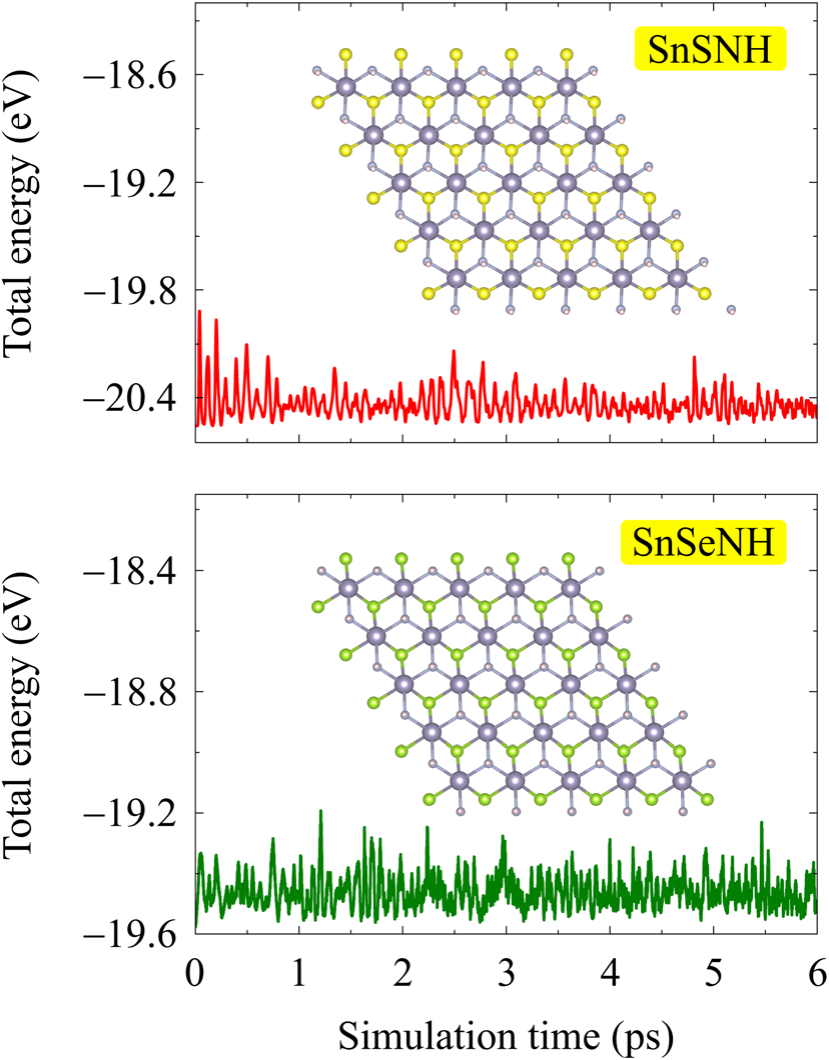
The time-dependent total energy fluctuations from AIMD simulations of SnSNH and SnSeNH monolayers. The inset figures show the corresponding atomic configurations after the simulations.

To assess thermodynamic stability, AIMD simulations are performed. The variation of total energy with simulation time, together with the resulting atomic configurations of SnSNH and SnSeNH structures are presented in Fig. 3. The system shows negligible total energy fluctuations throughout the 6 ps at 300 K, indicating good thermal equilibrium. At the end of the simulation, the structural frameworks are preserved and no bond dissociation observed. This demonstrates that the SnSNH and SnSeNH monolayers are thermally stable at 300 K. However, although the structures remain stable at 300 K throughout the simulation, the 6 ps trajectory is relatively short. Consequently, these results should be regarded as a preliminary indication of room-temperature stability rather than definitive proof.

### 3.2 Mechanical properties

The mechanical stability of SnSNH and SnSeNH monolayers is evaluated by examining their elastic response under in-plane deformation. Small biaxial strains between −1.5 and 1.5% are applied, the corresponding strain–energy relationships are analyzed. These energy variations are subsequently fitted to determine the elastic stiffness coefficients, namely *C*_11_, *C*_12_, *C*_22_, and *C*_66_.^34^ Because the SnSNH and SnSeNH monolayers crystallize in a hexagonal structure, their elastic tensors can be simplified, leaving only two independent constants, *C*_11_ and *C*_12_. The remaining components are related through *C*_66_ = (*C*_11_ − *C*_12_)/2 and *C*_22_ = *C*_11_. As presented in Table 2, the two investigated SnSNH and SnSeNH monolayers exhibit positive elastic constants, confirming their elastic stability. Specifically, the calculated values of *C*_11_/*C*_12_/*C*_66_ of SnSNH and SnSeNH are 91.00/23.85/33.58 N m^−1^ and 76.45/25.02/25.72 N m^−1^, respectively. It can be observed that the requirement of *C*_11_ > *C*_12_ is satisfied for these two structures, ensuring compliance with the Born–Huang mechanical stability criterion.^35,36^ These findings verify that SnSNH and SnSeNH monolayers are mechanically stable and promising for experimental fabrication.

**Table 2:** Elastic coefficients *C*_*ij*_ (N m^−1^), Young's modulus *Y*_2D_ (N m^−1^), Poisson's ratio *ν*, PBE/HSE06 bandgaps *E*_g_ (eV), work functions *Φ*_1_ and *Φ*_2_ (eV) at the chalcogen (S/Se) and H surfaces, respectively, and the vacuum level difference Δ*Φ* (eV) for the SnSNH and SnSeNH monolayers

	*C*_11_	*C*_12_	*C*_66_	*Y*_2D_	*ν*	*E* ^PBE^_g_	*E* ^HSE06^_g_	*Φ*_1_	*Φ*_2_	Δ*Φ*
SnSNH	91.00	23.85	33.58	84.75	0.26	1.33	2.13	6.53	3.35	3.18
SnSeNH	76.45	25.02	25.72	68.27	0.33	0.29	0.95	5.47	2.78	2.69

Additionally, according to the calculated elastic stiffness constants, the mechanical response of SnSNH and SnSeNH monolayers is further analyzed in terms of their stiffness and deformation behavior. This is achieved by calculating the 2D Young's modulus (*Y*_2D_) and Poisson's ratio (*ν*_2D_), defined as follows:2
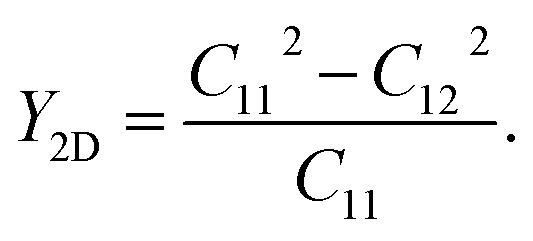
3
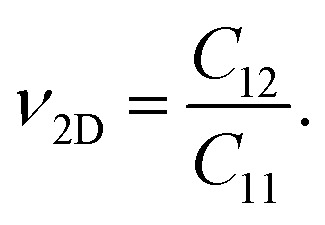


**Fig. 4:**
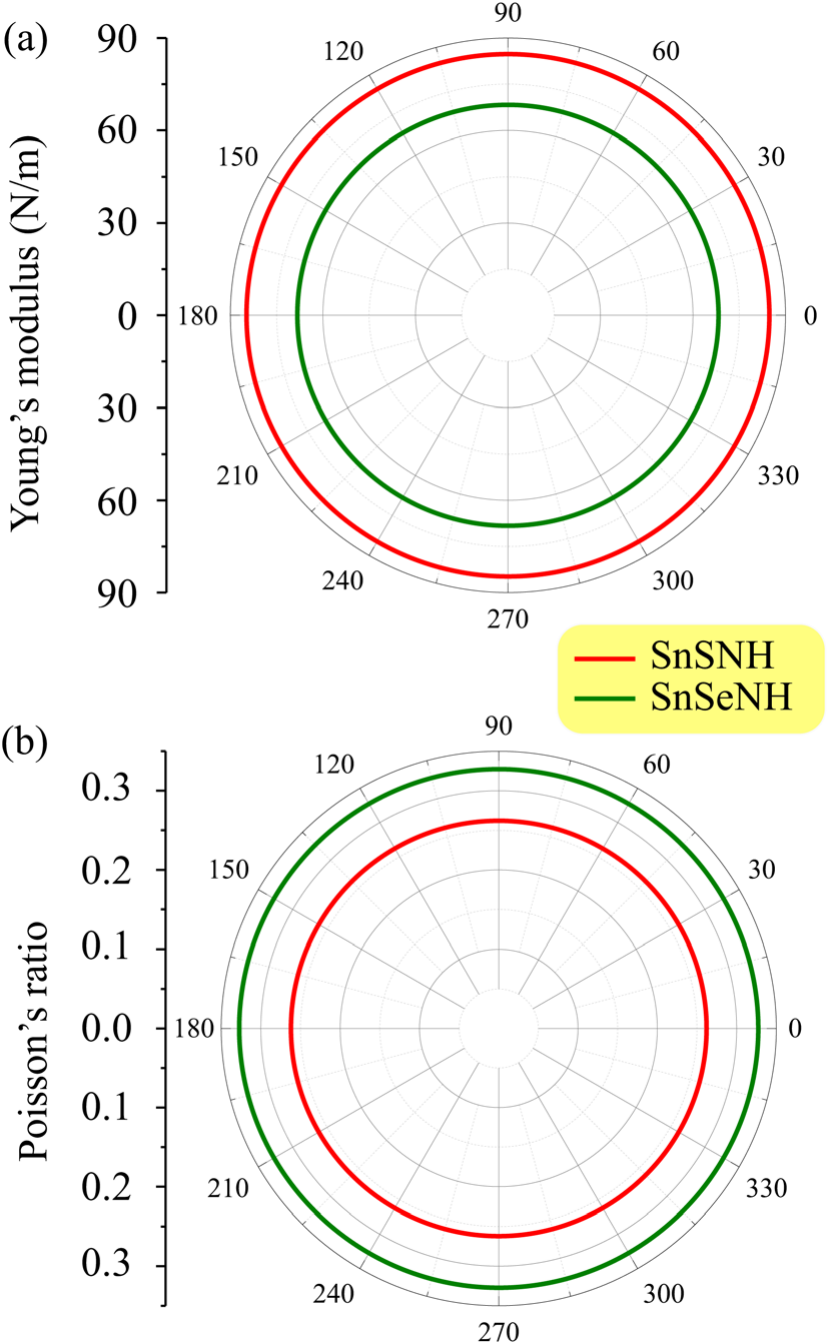
(a) Young's modulus and (b) Poisson's ratio of SnSNH and SnSeNH as a function of the orientation angle.

The directional dependences of *Y*_2D_ and *ν*_2D_ are depicted in Fig. 4. The circular symmetry of the plots clearly indicates that both *Y*_2D_ and *ν*_2D_ are independent of direction, confirming isotropic elastic behavior. The SnSNH monolayer possesses high *Y*_2D_ of 84.75 N m^−1^, while the SnSeNH exhibits lower *Y*_2D_ of 68.27 N m^−1^. This decreasing trend is associated with the increasing atomic radius of the chalcogen atoms. In contrast to other 2D systems such as graphene (*Y*_2D_ = 336 N m^−1^),^37^ MoSi_2_P_4_ (*Y*_2D_ = 203.00 N m^−1^),^38^ and ZrGeN_3_H (*Y*_2D_ = 217.88 N m^−1^),^39^ the SnSNH (*Y*_2D_ = 84.75 N m^−1^) and SnSeNH (*Y*_2D_ = 68.27 N m^−1^) monolayers exhibit significantly lower stiffness. While this reduced Young's modulus indicates enhanced mechanical flexibility, rendering them highly promising for flexible, bendable, and wearable nanoelectronics, it also presents a practical limitation with respect to mechanical stability. Specifically, highly compliant 2D materials are more susceptible to structural deformations. Consequently, the practical integration of SnSNH and SnSeNH monolayers into physical devices may necessitate careful handling and low-stress fabrication protocols. The Poisson's ratio shows a slight increase from 0.26 for SnSNH to 0.33 for SnSeNH as listed in Table 1. These values are comparable with 2D materials of ZrSeTe (*ν*_2D_ = 0.26),^40^ PtSeTe (*ν*_2D_ = 0.27),^41^ and MoS_2_ (*ν*_2D_ = 0.25),^42^ implying the good mechanical stability under in-plane strains of the SnSNH and SnSeNH monolayers.

### 3.3 Electronic properties

**Fig. 5:**
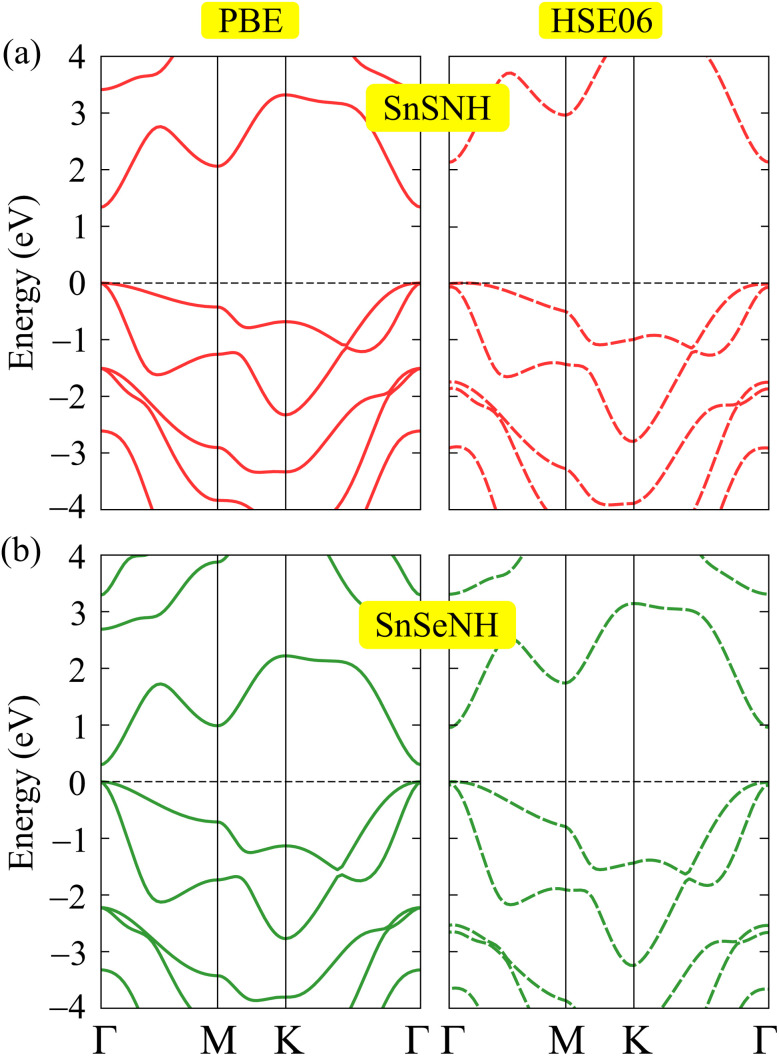
Band structures of (a) SnSNH and (b) SnSeNH calculated using PBE and HSE06 functionals.

The investigation of electronic properties is essential for understanding and predicting the performance of materials in advanced technological applications, including nanoelectronic devices, optoelectronic systems, and energy conversion. Accordingly, the electronic characteristics of SnSNH and SnSeNH monolayers are systematically investigated in this section. Fig. 5 illustrates the band structures of SnSNH and SnSeNH, calculated along the *Γ*–*M*–*K*–*Γ* path in the Brillouin zone (BZ). At equilibrium, the two SnSNH and SnSeNH monolayers behave as direct semiconductors, with the valence band maximum (VBM) and conduction band minimum (CBM) simultaneously located at the *Γ* point. As detailed in Table 1, the standard PBE functional predicts bandgaps of 1.33 eV for SnSNH and 0.29 eV for SnSeNH. This reduction can be attributed to the lower electronegativity and more extended atomic orbitals of Se compared to S, which broadens the energy bands and narrows the gap. To correct for the well-known bandgap underestimation inherent to standard DFT functionals, the HSE06 hybrid functional was employed. The HSE06 calculations confirm the direct bandgap nature while predicting more accurate, enlarged bandgap values of 2.13 eV and 0.95 eV for the SnSNH and SnSeNH monolayers, respectively.

Furthermore, the work functions (*Φ*) of the SnSNH and SnSeNH configurations are evaluated to assess the ability of electrons to escape from the material surfaces. The work function is defined as the energy difference between the vacuum potential (*E*_vac_) and the Fermi level (*E*_F_):

4 *Φ* = *E*_vac_ − *E*_F_.

The structural asymmetry of the Janus monolayer, arising from the differing electronegativities of the distinct top and bottom atomic layers, induces an asymmetric out-of-plane electron charge density distribution. This localized charge imbalance manifests as a macroscopic electrostatic potential difference between the two surfaces. Consequently, this potential gradient establishes a strong intrinsic built-in electric field *E*_int_ directed perpendicular to the monolayer plane. This built-in electric field breaks the out-of-plane structural inversion symmetry and is fundamentally responsible for driving the unique electronic properties of the Janus structure.

**Fig. 6:**
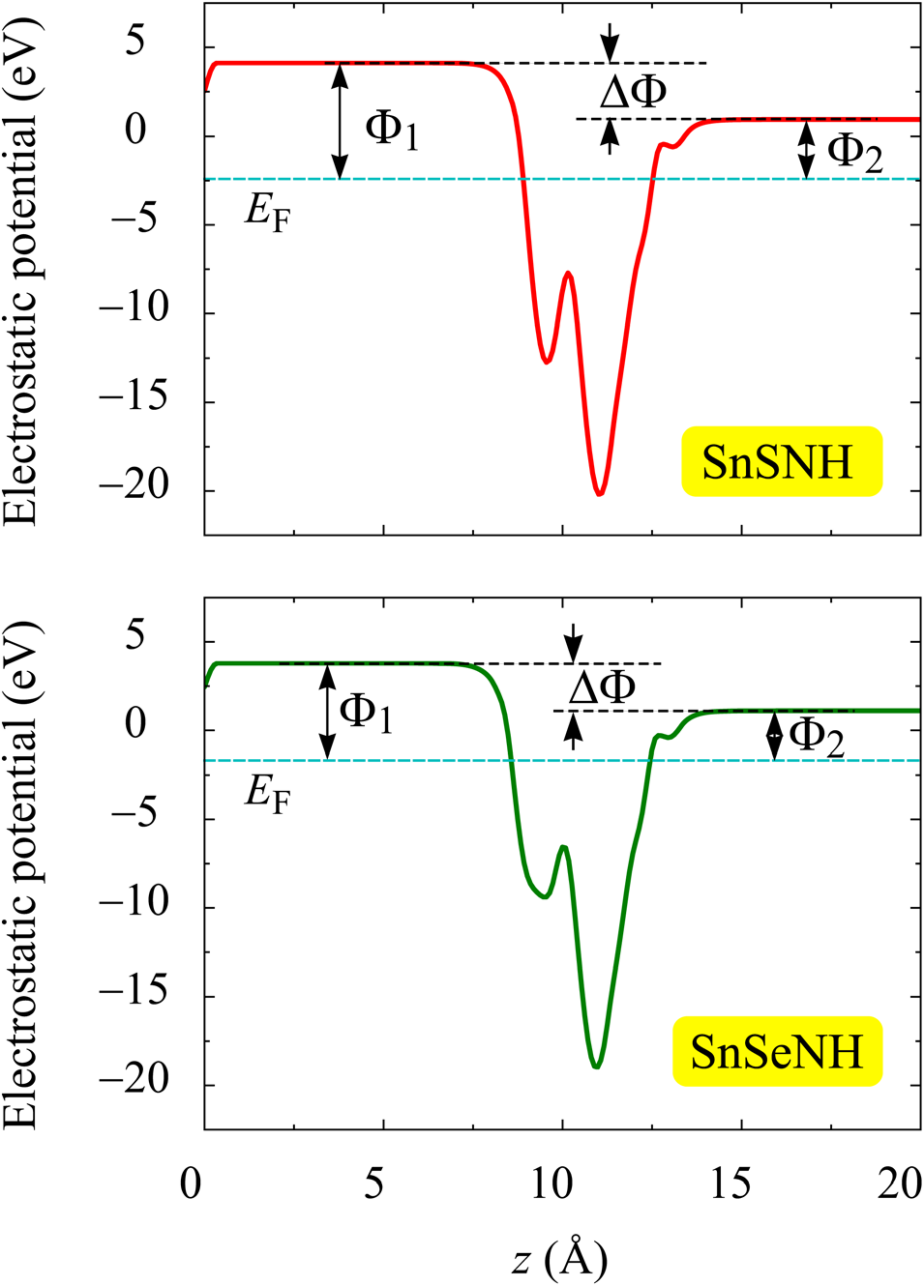
Electrostatic potentials of the SnSNH and SnSeNH monolayers along the *z*-direction. The Fermi level (*E*_F_) is denoted by the horizontal dashed line. *Φ*_1_ and *Φ*_2_ represent the work functions at the chalcogen (S/Se) and hydrogenated surfaces, respectively.

Fig. 6 presents the calculated planar-averaged electrostatic potentials used to extract the work function values. The results reveal a decreasing trend in the work function from SnSNH to SnSeNH. This behavior implies that electron emission is more favorable for SnSeNH. The calculated Δ*Φ* values decrease from 3.18 eV for SnSNH to 2.69 eV for SnSeNH, as summarized in Table 2. As presented in Table 2, the calculated work function *Φ*_1_ at the chalcogen (S/Se) surface is significantly larger than the work function *Φ*_2_ at the hydrogenated (H) surface. This substantial energy difference indicates a lower emission barrier at the hydrogenated side, implying that electrons are emitted more readily from the H surface. Physically, this pronounced inequality arises from a strong intrinsic out-of-plane dipole moment induced by the asymmetric structure of these Janus materials, which generates a robust built-in electric field directed across the monolayer. For photocatalytic applications, it provides a strong driving force that facilitates the efficient spatial separation of photogenerated charge carriers, ultimately mitigating electron–hole recombination.

### 3.4 Transport properties

Moreover, to further evaluate the suitability for electronic applications of SnSNH and SnSeNH monolayers, their charge transport properties are analyzed. In particular, the carrier mobility *µ*_2D_ and its associated parameters are calculated using the deformation potential (DP) theory, as proposed by Bardeen and Shockley:^25,43^5
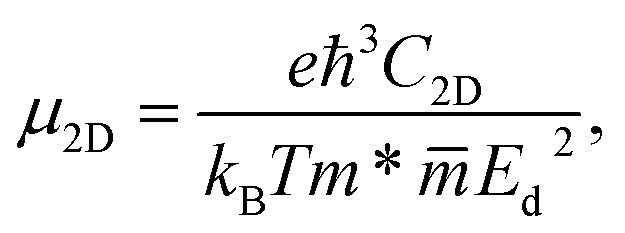
where *k*_B_, *ℏ*, *e*, and *T* denote the Boltzmann constant, reduced Planck constant, elementary charge, and the operating temperature of 300 K, respectively. 
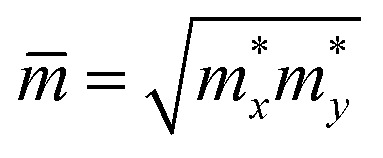
 represents the averaged effective mass. Meanwhile, m*, *C*_2D_, and *E*_d_ correspond to the carrier effective mass, the in-plane elastic modulus derived from the strain–energy relationship, and the deformation potential constant obtained from the variation of band-edge energies under strain, respectively. The parameters *C*_2D_ and *E*_d_ are determined according to the following expressions:^43^6
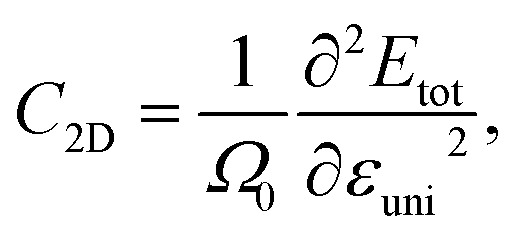
7
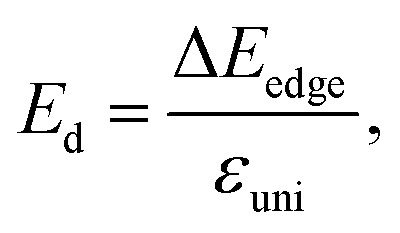
where *E*_tot_ is the total energy of the system, *Ω*_0_ denotes the equilibrium area of the optimized unit cell, and *ε*_uni_ represents the applied uniaxial strain along the in-plane directions. The quantity Δ*E*_edge_ indicates the shift in the conduction or valence band edges induced by the applied strain.

**Fig. 7:**
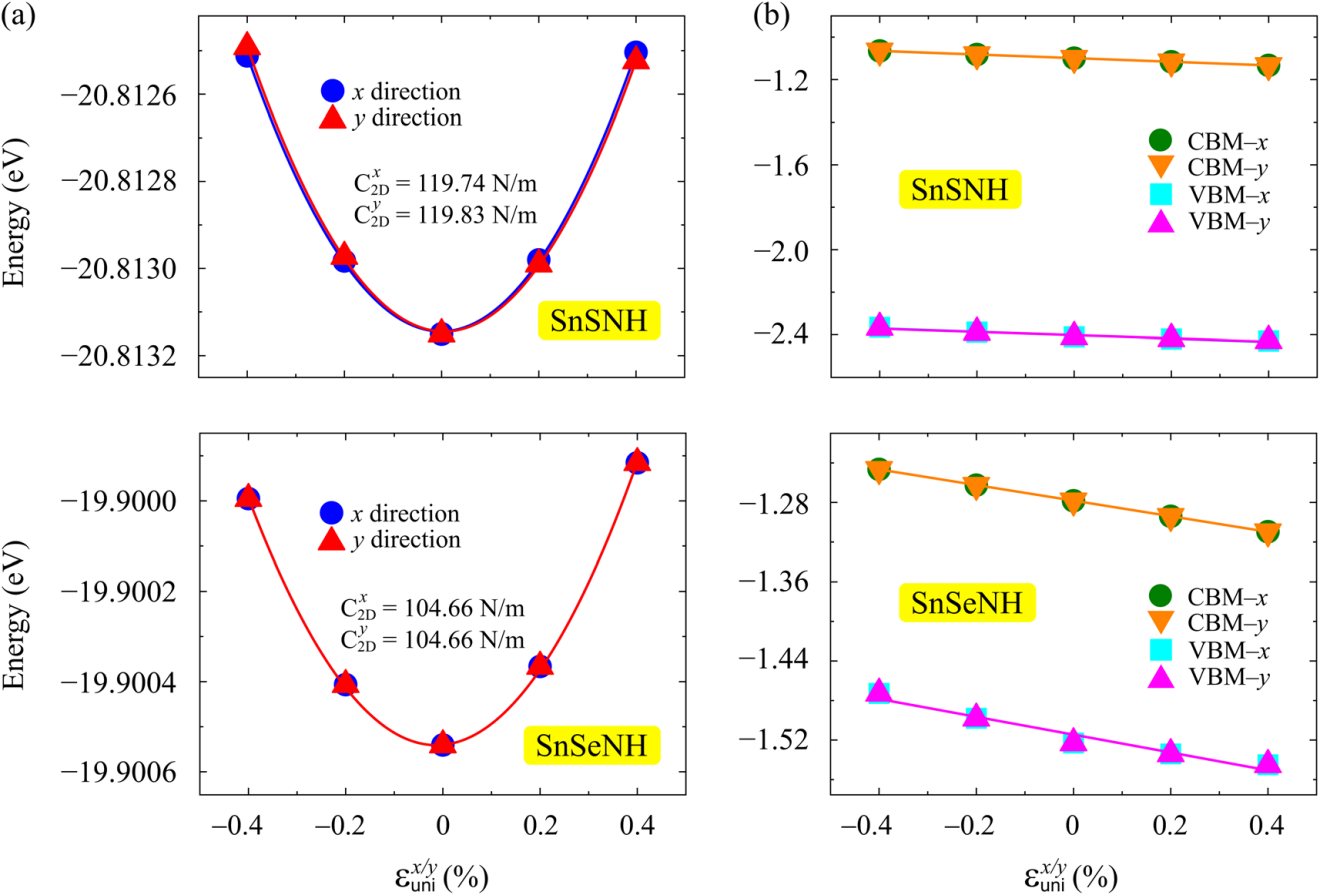
Calculated (a) total energy and (b) band-edge positions of SnSNH and SnSeNH systems *versus* uniaxial strains.

To calculate the elastic modulus *C*_2D_ and the deformation potential constant *E*_d_, uniaxial strains *ε*_uni_^*x*/*y*^ from −0.4 to 0.4% are applied along the *x*/*y* directions. The strain-dependent variations of the VBM and CBM are fitted using established approaches^43,44^ to extract the band-edge positions and total energy changes of the SnSNH and SnSeNH monolayers, as depicted in Fig. 7. To validate the extraction of the deformation potential constants, the coefficient of determination *R*^2^ was calculated for all linear fits of the band-edge shifts *versus* applied strain, yielding values greater than 0.99 in both studied monolayers that confirm the deformation strictly remains within the linear elastic regime. The results depict that the band-edge energies and elastic modulus *C*_2D_ are nearly identical along both in-plane directions for the two systems. A slight variation in the total energy is observed for the SnSNH structure. The calculated *C*_2D_ and *E*_d_ values are listed in Table 3. The SnSeNH exhibits directionally isotropic deformation potential constants of −7.88 eV for electron and −9.02 eV for hole. Meanwhile, the *E*_d_ values of SnSNH are directional anisotropy. Similar anisotropic behavior of *E*_d_ has also been reported in other 2D materials such as STiSiAs_2_ (ref. 33) and Ga_2_Te_3_.^45^

**Table 3:** Calculated transport properties of SnSNH and SnSeNH monolayers along the *x* and *y* directions: effective masses m*/*m*_0_, 2D elastic moduli *C*_2D_ (N m^−1^), deformation potential constants *E*_d_ (eV), and carrier mobilities *µ* (cm^2^ V^−1^ s^−1^). *m*_0_ is the free electron mass

		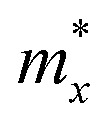	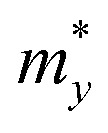	*C*_2D_^*x*^	*C*_2D_^*y*^	*E*_d_^*x*^	*E*_d_^*y*^	*µ*_*x*_	*µ*_*y*_
Electron	SnSNH	0.29	0.30	119.74	119.83	−8.52	−8.55	410.23	394.51
	SnSeNH	0.26	0.26	104.66	104.66	−7.88	−7.88	530.37	530.37
Hole	SnSNH	3.51	2.48	119.74	119.83	−8.04	−7.60	3.81	6.04
	SnSeNH	1.59	1.31	104.66	104.66	−9.02	−9.02	11.94	14.49

The carrier effective mass m* is another fundamental parameter that significantly influences charge transport and mobility in semiconductor materials. It can be derived from the curvature of the electronic band structure near the band edges according to the following expression:^46^8
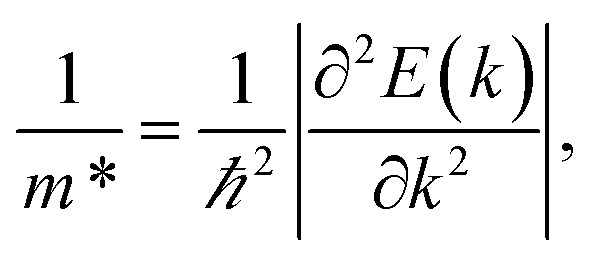
where *E*(*k*) represents the energy dispersion at the band edges with respect to the wave vector *k*. The calculated effective masses of carriers along the *x* and *y* axes are anisotropic as listed in Table 3. Generally, the electron effective masses 
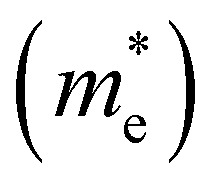
 of SnSNH and SnSeNH monolayers are smaller than their hole effective masses 
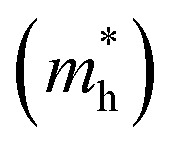
. The SnSNH has the highest 
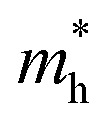
 of 3.51 *m*_0_ along *x* and 2.48 *m*_0_ along *y* direction, whereas the SnSeNH possesses the lowest 
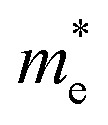
 values of 0.26 *m*_0_ for both *x* and *y* directions. Consequently, electrons are expected to respond more readily to external electric fields than holes along the two in-plane directions. It should be noted that the deformation-potential formalism accounts only for acoustic-phonon-limited transport and neglects other scattering mechanisms, such as polar optical phonon and impurity scattering. Therefore, the calculated carrier mobilities represent ideal intrinsic upper-bound estimates.

By substituting the calculated *E*_d_, *C*_2D_, and m* into eqn (5), the carrier mobilities of SnSNH and SnSeNH monolayers can be obtained. For hole transport, the SnSNH exhibits lower mobility, which can be attributed to its relatively large effective mass, as discussed previously. In contrast, the SnSNH and SnSeNH monolayers demonstrate high carrier electron mobilities, reaching 410.23 and 530.37 cm^2^ V^−1^ s^−1^, respectively. These obtained mobility values are higher than other reported 2D materials such as MoS_2_ (∼200 cm^2^ V^−1^ s^−1^),^47^ SWGeN_2_ (205 cm^2^ V^−1^ s^−1^)^48^ or WSi_2_N_4_ (119 cm^2^ V^−1^ s^−1^),^49^ and SMoSiP_2_ (306.52 cm^2^ V^−1^ s^−1^).^50^ The findings reveal that the carrier mobilities in SnSNH are direction-dependent and differ significantly between electrons and holes. This confirms its anisotropic transport behavior, which enhance the performance and design flexibility of advanced electronic, optoelectronic, and energy devices.

### 3.5 Photocatalytic properties

Lastly, the capability of SnSNH and SnSeNH monolayers to act as photocatalysts for water splitting is examined. It is well known that the water splitting process consists of fundamental reactions: hydrogen evolution reaction, described by 2H^+^ + 2e^−^ → H_2_ and the oxygen evolution reaction, expressed as H_2_O + 2h^+^ → 
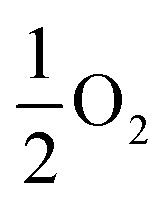
 + 2H^+^. The effective semiconductor photocatalysts need to satisfy two main conditions. The first is moderate band gaps for absorbing the visible spectrum but must be larger than the thermodynamic requirement of 1.23 eV. The second is appropriate band edge positions straddle the redox potentials of water, in which at pH = 0, the CBM lies above the H^+^/H_2_ reduction potential of −4.44 eV and the VBM lies below the O_2_/H_2_O oxidation potential of −5.67 eV. The band edge positions of the SnSNH and SnSeNH monolayers are calculated based on the Mulliken electronegativity equation, as follows:^51^

9 *E*_VBM_ = *χ* − *E*_e_ + 0.5 *E*_g_.

10 *E*_CBM_ = *E*_VBM_ − *E*_g_.

where, *E*_g_, *E*_e_ and *χ* refer to the bandgap energy of the material, the free-electron energy on the hydrogen scale, and the average electronegativity, respectively.

**Fig. 8:**
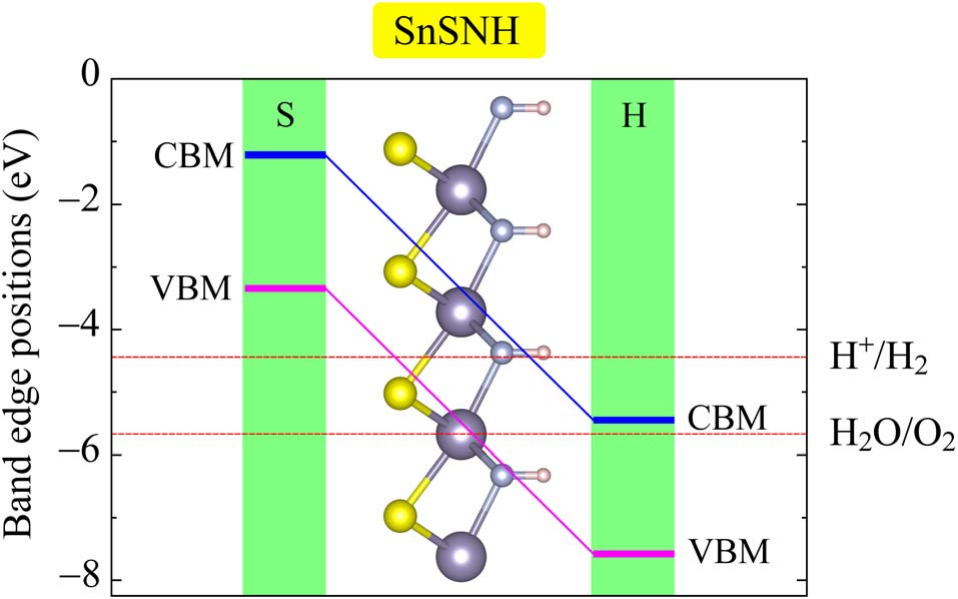
Calculated band edge positions of Janus SnSNH monolayer relative to the vacuum level. The horizontal dashed lines represent the standard redox potentials for water splitting at pH = 0.

From Fig. 6, we can observe the differences of electrostatic potentials *Φ*_1_ and *Φ*_2_ of X and H layers, respectively. Consequently, the CBM position of X layer is more positive than the H layer, and the VBM position of H layer is more negative than the X layer. Based on the band gap requirements, only the SnSNH monolayer qualifies for water splitting applications. The corresponding band edge alignments of SnSNH at pH = 0 are presented in Fig. 8. It should be noted that the band edge alignments evaluated in this study are based on standard state conditions (pH = 0). Our primary objective is to establish a fundamental theoretical baseline regarding the intrinsic electronic properties and photocatalytic viability of the novel Janus architecture. However, we acknowledge that practical experimental water splitting is frequently conducted under neutral or near-neutral conditions, where the pH-dependence of redox potentials becomes a critical factor for physical device implementation. As shown in Fig. 8, the CBM of S layer has higher energy than the reduction potential and the VBM of H layer is lower energy than the oxidation potential. Thus, the photogenerated electrons and holes can easily move from conduction and valence bands for the hydrogen evolution reaction and oxygen evolution reaction, respectively. In addition, with a moderate band gap of 2.13 eV at HSE06 level, the SnSNH is found as a potential photocatalytic material for overall water splitting. It should be noted that the bifunctional photocatalytic behavior fundamentally originates from the intrinsic structural asymmetry of the Janus monolayer, rather than from a claim of having fully characterized the kinetic reaction pathways. With a calculated narrow bandgap of 0.95 eV, SnSeNH does not meet the minimum thermodynamic requirement of 1.23 eV necessary to drive overall water splitting. Consequently, its photocatalytic utility as an isolated monolayer is restricted to half-reactions, specifically the hydrogen evolution reaction. To operate effectively and prevent rapid electron–hole recombination, the system would necessitate the introduction of sacrificial electron donors to rapidly scavenge photogenerated holes.

## 4 Conclusion

In conclusion, we have employed first-principles DFT method to design and investigate the structural and dynamical properties of SnXNH monolayers, where X corresponds to the chalcogen atoms S, Se, and Te. Phonon spectra reveal that SnTeNH is dynamically unstable due to the presence of imaginary modes, whereas SnSNH and SnSeNH show robust dynamical stability. These two SnSNH and SnSeNH monolayers further exhibit excellent thermal stability at room temperature and favorable energetic and mechanical properties, suggesting their potential for experimental realization. Their electronic and transport characteristics are subsequently examined. The PBE and HSE06 functionals consistently indicate that SnSNH and SnSeNH are direct semiconductors with energy gaps ranging from 0.29 to 2.13 eV. The SnSNH possesses suitable bandgap enabling visible-light absorption suitable for harvesting the higher-energy portion of the visible spectrum. Transport properties calculated using the deformation potential theory show that the electron mobilities of SnSNH and SnSeNH monolayers are much higher than their hole mobilities related to the small effective masses. Furthermore, the mobilities of SnSNH exhibit clear anisotropy with respect to both carrier type and transport direction. Due to the large surface potential differences of S/Se and H layers, the band edge positions of SnSNH and SnSeNH satisfy the requirements for water redox potential. Hence, these results demonstrate the suitability of the Janus SnSNH and SnSeNH structures for electronic, photovolatic and photocatalytic applications.

## Conflicts of interest

There are no conflicts of interest to declare.

## Data Availability

The data that support the findings of this study are available from the authors upon reasonable request.
